# Transcriptomic Dissection of *Bothrops moojeni* Venom Reveals Fraction-Specific Modulation of Host Cellular Pathways

**DOI:** 10.3390/ijms27135943

**Published:** 2026-07-01

**Authors:** Fernanda D’Amélio, Rodrigo Pinheiros Araldi, Isabel de Fátima Correia Batista, Álvaro Rossan de Brandão Prieto-da-Silva, Irina Kerkis

**Affiliations:** 1 Postgraduate Program in Structural and Functional Biology, UNIFESP, São Paulo 04023-062, Brazil; alvaro.prieto@butantan.gov.br; 2 Centre of Excellence in New Target Discovery (CENTD), Butantan Institute, São Paulo 05503-900, Brazil; 3 Laboratory of Genetics, Butantan Institute, São Paulo 05503-900, Brazil; isabel.batista@butantan.gov.br; 4 BioDecision Analytics Ltd., São Paulo 01451-917, Brazil; rodrigo.araldi@biodecisionanalytics.com

**Keywords:** *Bothrops moojeni* venom, OC, transcriptomics, venom fractions, bone remodeling, metalloproteinases, phospholipases A_2_, venom-derived therapeutics, immune signaling, extracellular matrix

## Abstract

Snake venom is a remarkably complex cocktail of bioactive molecules capable of hijacking diverse host physiological processes, yet how individual venom components drive these cellular responses remains a bit of a black box. To map these dynamics, we ran a comparative transcriptomic analysis on human osteoclastogenic cultures, exposing them continuously to crude *Bothrops moojeni* venom and its high (HMM) and low (LMM) molecular mass fractions throughout differentiation. This allowed us to capture the cumulative transcriptional shifts that unfold across the entire lifecycle of osteoclast development. The crude venom triggered a sweeping response, deeply impacting neuroimmune, extracellular matrix remodeling, inflammatory, and apoptotic pathways—reflecting a massive reshuffling of cellular regulatory networks. When we looked at the fractions, clear dividing lines emerged. The HMM fraction, packed with metalloproteinases and serine proteases, mostly drove pathways tied to cytoskeletal remodeling, intracellular trafficking, and osteoclast-associated signaling. In contrast, the LMM fraction—home to phospholipases A_2_, disintegrins, and small peptides—steered a much more targeted course, influencing immune regulation, proliferative signaling, and metabolic homeostasis while noticeably turning down catalytic and binding functions. Interestingly, all venom-treated groups shared a drop-off in ATP-dependent and ligand-binding categories, pointing to a common disruption in core metabolic and signaling processes. Taken together, these findings offer a clearer mechanistic look at how different *B. moojeni* venom components target bone remodeling pathways, highlighting the power of transcriptomics for untangling complex venom–host interactions.

## 1. Introduction

Snake venoms are among the most intricate and evolutionarily refined biochemical systems in nature, capable of modulating a broad spectrum of physiological processes through highly diverse bioactive components [1,2,3,4]. Among them, the venom of *Bothrops moojeni*—a medically important pit viper endemic to South America—has garnered increasing attention not only for its potent cytotoxic and proinflammatory activities, but also for its emerging therapeutic potential. Composed of a dynamic mixture of bioactive proteins and peptides, including metalloproteinases (SVMPs), phospholipases A_2_ (PLA2s), disintegrins, and cysteine-knot peptides, *B. moojeni* venom interacts with multiple cellular systems, influencing coagulation, immunity, tissue remodeling, and bone metabolism [5,6].

While the toxicological effects of crude *B. moojeni* venom are relatively well-characterized, the specific molecular and transcriptomic responses elicited by its individual components remain poorly understood [6,7,8,9]. Fractionation of crude venom into high molecular mass (HMM) and low molecular mass (LMM) components provides an effective strategy to dissect the discrete biological activities of its constituents. Previous studies, including our own [10], suggest that the LMM fraction—enriched in disintegrins and small peptides—modulates osteoclastogenesis and cytokine signaling with comparatively lower enrichment of inflammatory damage. In contrast, the HMM fraction, dominated by metalloproteinases and serine proteases, has been associated with the enrichment of proinflammatory and bone-resorption-related pathways, suggesting distinct and potentially complementary roles in skeletal pathology.

In this study, we present a comprehensive transcriptomic and pathway-level comparison of crude and fractionated *B. moojeni* venom during human OC differentiation. Using a continuous exposure model spanning OC commitment and maturation, we captured the cumulative transcriptomic alterations induced by each venom preparation during OC differentiation. This approach enabled a direct comparison of gene expression patterns and functional enrichment in Gene Ontology categories and signaling pathways, providing mechanistic insights into venom–host interactions.

Our analysis reveals that crude venom broadly modulates cellular differentiation, immune signaling, and metabolic processes. Notably, the HMM fraction was associated with the enrichment of intracellular trafficking and proinflammatory and bone-resorptive related pathways, whereas the LMM fraction predominantly affected homeostatic and immunomodulatory networks with a comparatively lower enrichment of inflammatory signaling. These distinct transcriptomic signatures highlight the functional compartmentalization of venom components and underscore their differential influence on OC-associated cellular pathway.

By elucidating the fraction-specific molecular effects of *B. moojeni* venom on osteoclastogenic cultures, this study advances a systems-level understanding of venom bioactivity and provides a framework for future translational investigations in bone remodeling, osteoimmune disorders, and venom-derived therapeutic discovery. The identification of candidate pathways and molecular targets may support future strategies in bone regeneration and bioactive compound discovery.

## 2. Results

**Figure 1:**
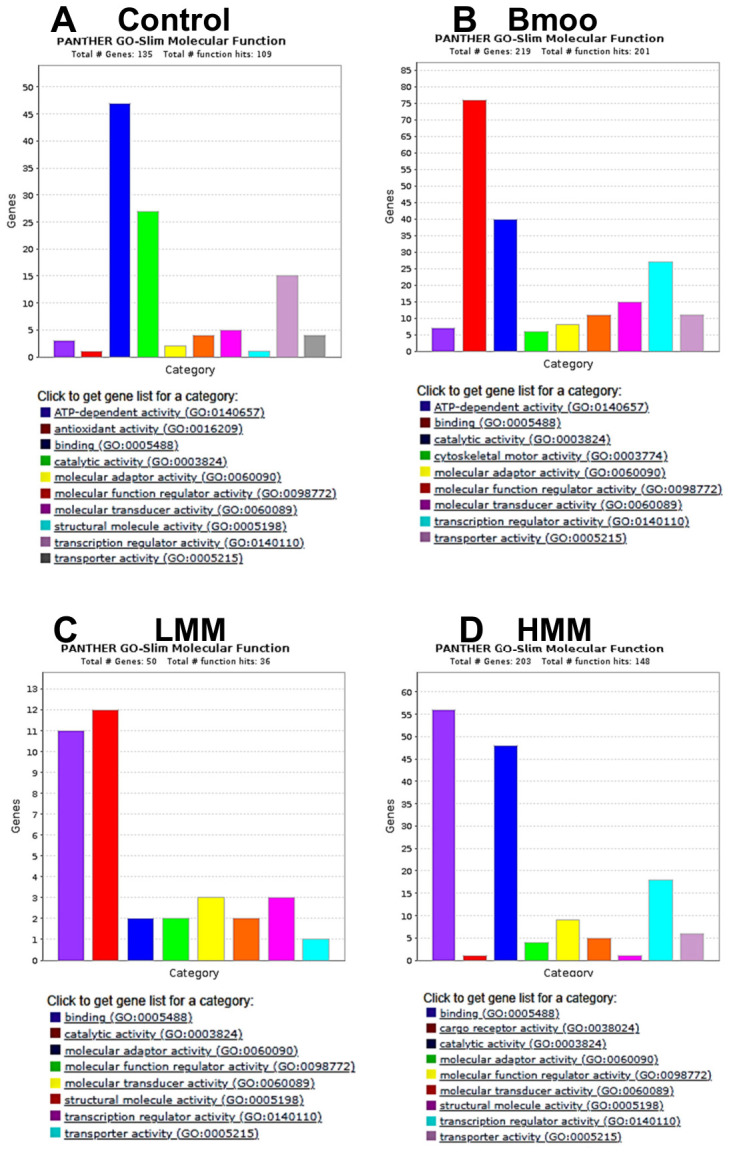
**Molecular function enrichment analysis.** (**A**) Control group; (**B**) Group treated with *Bothrops moojeni* venom; (**C**) Group treated with the low molecular mass fraction; (**D**) Group treated with the high molecular mass fraction. Analysis was performed using Panther Classification System software, version 19.0, highlighting significantly enriched biological pathways influenced by the treatments.

Functional enrichment analysis of genes exclusively expressed in each experimental group identified distinct molecular responses to crude and fractionated *B. moojeni* venom exposure. Within the Molecular Function (MF) domain, the control group exhibited enrichment of antioxidant activity and structural molecular function categories that were entirely absent from all venom-treated groups (crude venom, HMM, or LMM fractions). Across all treated groups, ATP-dependent activities and ligand binding function categories showed reduced representation compared with the control condition. Notably, the HMM group demonstrated selective enrichment of genes associated with cargo receptor activity (Figure 1).

**Figure 2:**
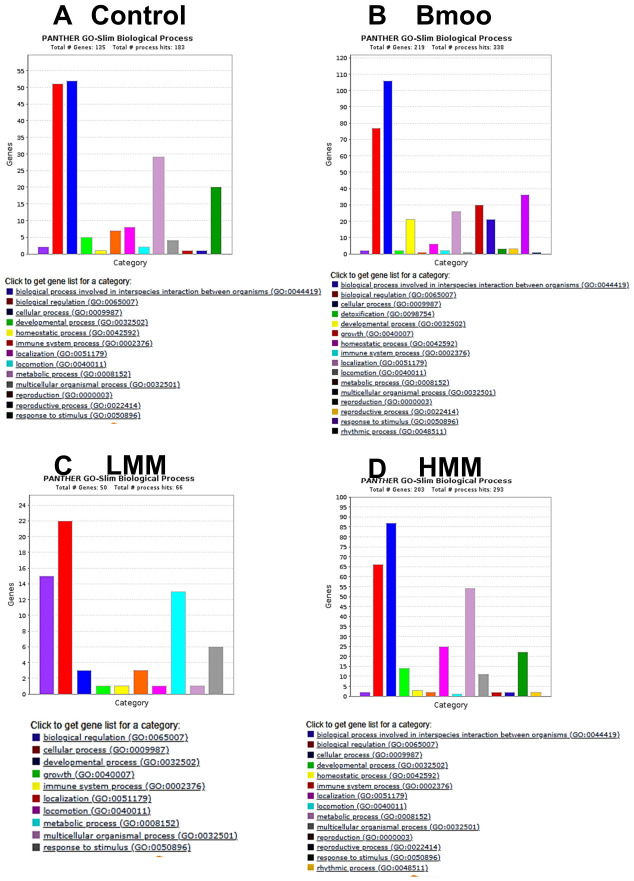
Biological process enrichment analysis. (**A**) Control group; (**B**) Group treated with *Bothrops moojeni* venom; (**C**) Group treated with the low molecular mass fraction; (**D**) Group treated with the high molecular mass fraction. Analysis was performed using Panther Classification System software, version 19.0, highlighting significantly enriched biological pathways influenced by the treatments.

In the Biological Process (BP) analysis, no unique enrichment was identified in the control group. Crude venom treatment was characterized by the enrichment of detoxification, rhythmic, and cell growth-related processes (Figure 2). The HMM fraction also showed the modulation of rhythmic processes. Conversely, the LMM group displayed a lower enrichment of the homeostasis-, reproduction-, and interspecies interaction-associated pathways while maintaining enrichment in growth-associated processes.

**Figure 3:**
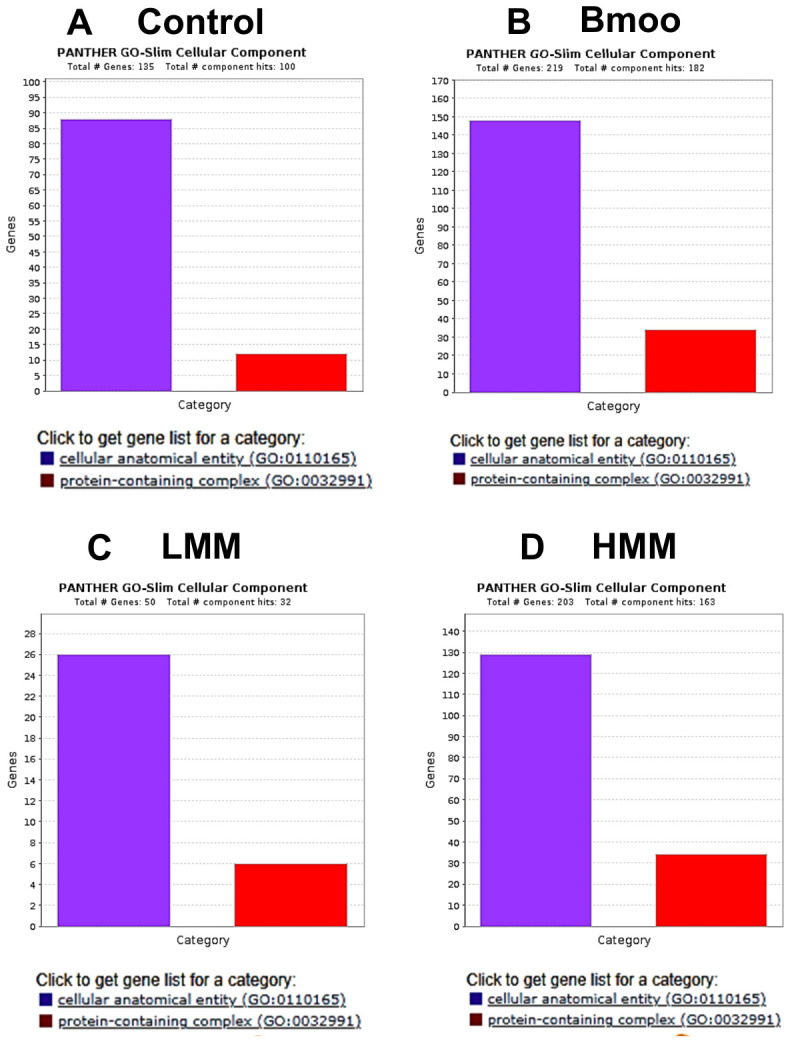
**Cellular component enrichment analysis.** (**A**) Control group; (**B**) Group treated with *Bothrops moojeni* venom; (**C**) Group treated with the low molecular mass fraction; (**D**) Group treated with the high molecular mass fraction. Analysis was performed using Panther Classification System software, version 19.0, highlighting significantly enriched biological pathways influenced by the treatments.

The Cellular Component (CC) enrichment profiles were largely conserved across all experimental groups. Categories associated with core structural elements and protein complexes appeared consistently across treatments, revealing a highly uniform distribution among the group-exclusive gene sets (Figure 3).

**Figure 4:**
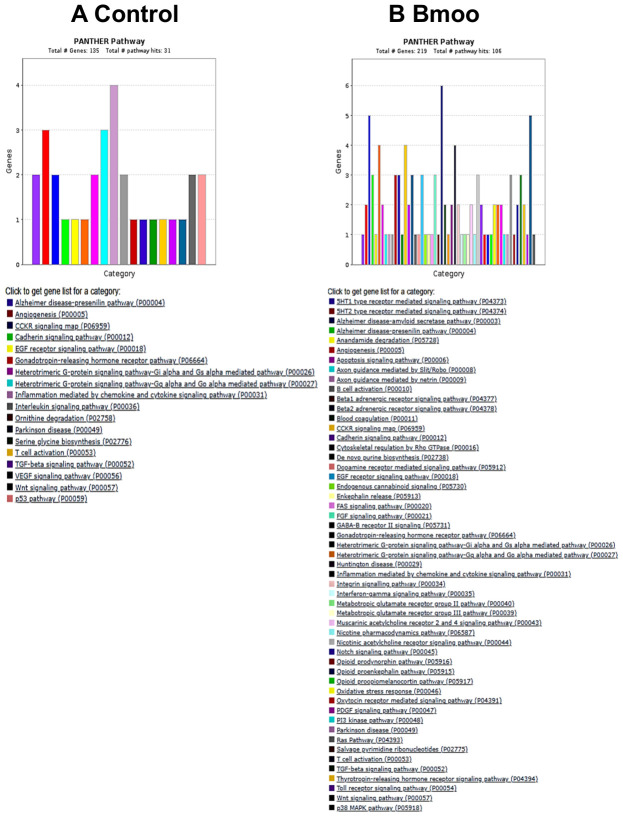
**Pathway enrichment analysis.** (**A**) Pathway enrichment results for the control group. (**B**) Pathway enrichment results for the group treated with *Bothrops moojeni* venom. The comparison highlights the venom-induced modulation of key biological pathways relative to the untreated condition. Analysis was performed using Panther Classification System software, version 19.0, highlighting significantly enriched biological pathways influenced by the treatments.

Pathway-level analysis revealed the most pronounced distinctions between the experimental groups. The control group’s exclusive gene set showed a unique enrichment of pathways related to interleukin signaling, ornithine degradation, glycine-serine biosynthesis, VEGF signaling, and p53-related regulatory responses (Figure 4A).

Exposure to crude *B. moojeni* venom was associated with a broad enrichment of pathways related to neuroimmune signaling, apoptosis, oxidative stress responses, integrin signaling, PI3K and Ras pathways, Toll-like receptor signaling, and p38 MAPK activation, alongside pathways tied to neurotransmission and neurodegenerative processes (Figure 4B). Notably, pathways involving coagulation, oxidative stress regulation, and Toll-like receptor-associated signaling were absent from this crude venom-exclusive gene profile.

**Figure 5:**
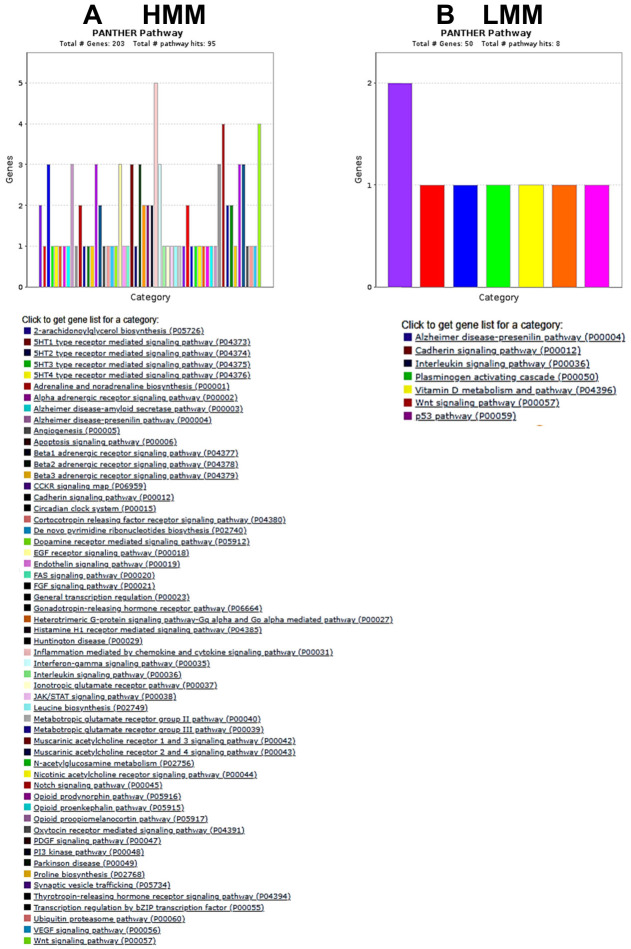
**Pathway enrichment analysis.** (**A**) Pathway enrichment results for osteoclasts treated with the high molecular mass (HMM) fraction of *Bothrops moojeni* venom. (**B**) Pathway enrichment results for osteoclasts treated with the low molecular mass (LMM) fraction of *Bothrops moojeni* venom. Analysis was performed using Panther Classification System software, version 19.0, highlighting significantly enriched biological pathways influenced by the treatments.

The HMM fraction was associated with the enrichment of pathways involving adrenergic and serotonergic signaling, circadian rhythm regulation, histamine and glutamate receptor activity, JAK/STAT signaling, ubiquitin-proteasome regulation, and synaptic vesicle trafficking (Figure 5A). Finally, the LMM fraction displayed a more selective enrichment profile, primarily centered on interleukin signaling, plasminogen activation, and the vitamin D metabolism pathways (Figure 5B). Notably, these specific functional profiles were completely absent from the crude venom and HMM fraction exclusive gene sets, highlighting the distinct molecular blueprint of the LMM components.

## 3. Discussion

Crude venom exposure broadly triggered wide-ranging transcriptional changes across multiple functional categories, particularly those tied to binding, catalytic activity, and molecular transduction. These shifts indicate a robust cellular response, including the activation of pathways involved in extracellular matrix (ECM) remodeling and signaling cascades associated with bone remodeling and osteoclast activity. Consistent with prior work, venom components like SVMPs and PLA_2_s were linked to the activation of inflammatory signaling pathways, including NF-κB, MAPK, and PI3K-AKT [6,10,11]. The enrichment of neuroimmune, apoptotic, and neurotransmission pathways further highlights how deeply crude venom impacts interconnected biological systems. Given this profile, crude *B. moojeni* venom provides a valuable experimental model for mapping complex disease pathways and pinpointing novel therapeutic targets (Table 1).

These specific inflammatory signaling processes are closely tied to osteoclastogenesis and bone-resorptive activity. In particular, mediators like IL-1β, IL-6, and prostaglandin-related signaling cascades are heavily implicated in managing immune–skeletal interactions and bone remodeling [12].

In contrast, the LMM fraction—which carries small bioactive molecules like disintegrins and cysteine-knot peptides—showed much less enrichment in categories related to transcription and catalytic activity. This profile likely reflects a more targeted regulatory effect compared to the sweeping transcriptional changes triggered by the crude venom and the HMM fraction.

The HMM fraction leaned more toward pathways regulating neuroendocrine signaling, intracellular trafficking, and protein turnover. Meanwhile, the LMM fraction uniquely favored immunomodulatory and osteotropic pathways, including vitamin D metabolism and plasminogen activation. These findings line up well with what we know about the pharmacological properties of venom-derived enzymes, peptides, and disintegrin-like molecules [13,14]. Taken together, the contrasting transcriptomic blueprints left by the HMM and LMM fractions reinforce the idea of functional compartmentalization within the venom. This suggests that these separate components could serve as distinct, valuable toolkits of bioactive molecules for research into immune regulation, proteostasis, and bone disorders (Table 1).

In the control osteoclast culture, several vital biological pathways governing bone homeostasis and cellular regulation were significantly enriched, reflecting a balanced physiological baseline. Interleukin signaling pathways (including IL-6, IL-1, IL-7, and IL-23) are known to help modulate osteoclast differentiation, survival, and function through complex mechanisms involving RANK/RANKL signaling, STAT activation, and co-stimulatory immunoreceptor pathways [15,16,17]. The ornithine degradation pathway is also tied to osteoclast metabolism and differentiation; recent evidence links Nrf2 activation to the suppression of osteoclastogenesis by regulating iron and ornithine metabolism [18]. Additionally, the serine-glycine biosynthesis pathway proves essential for osteoclast maturation, where it supports epigenetic regulation via alpha-ketoglutarate-dependent histone demethylation to drive NFATc1 expression [19]. VEGF signaling aids osteoclast survival and activity, emphasizing its dual role in both angiogenesis and bone remodeling [20,21]. Finally, the p53 tumor suppressor acts as a negative regulator of osteoclastogenesis by influencing autophagy and working alongside osteoprotegerin-mediated pathways to prevent runaway bone resorption [18,22]. Together, this intricate web of pathways highlights the sophisticated regulatory networks required to maintain healthy bone homeostasis under normal physiological conditions.

Across all venom-treated groups, the reduced enrichment of ATP-dependent and ligand-binding functional categories points to a shared alteration in metabolic and signaling networks that govern cellular communication and homeostasis. The exclusive enrichment of cargo receptor activity in the HMM group further suggests the modulation of intracellular transport-related pathways, potentially involving protein trafficking and autophagy-associated processes. Together, these findings reveal both common and fraction-specific transcriptomic responses to venom exposure, providing insight into cellular pathways differentially affected by the venom components.

Importantly, this study provides a molecular framework for understanding the differential cellular effects associated with crude and fractionated venom exposure. The identification of distinct pathway signatures may support future efforts aimed at isolating pharmacologically relevant molecules capable of modulating osteoclast-associated processes in bone disorders such as osteoporosis.

We acknowledge that our study is limited to transcriptomic enrichment analysis and lacks direct functional validation. However, the statistically robust enrichment patterns observed across multiple biological domains support the existence of distinct, venom-associated molecular responses. Integrating downstream functional assays and in vivo models will be important to validate the mechanistic hypotheses proposed here and to fully explore the therapeutic potential of venom-derived compounds in bone remodeling and related pathologies.

## 4. Methods and Materials

### 4.1. PBMCS Isolation and Differentiation

PBMCs were isolated using density gradient centrifugation with Ficoll–Paque (1.077 g/mL, Sigma-Aldrich, St. Louis, MO, USA). Approximately 20 mL of whole blood was collected from healthy male volunteers (aged 25–40 years) via venipuncture of the cubital fossa, in accordance with institutional ethical guidelines (Plataforma Brasil/CEP 1.806.596). The blood was diluted 1:1 in 0.9% saline solution, layered over Ficoll–Paque at a 1:3 ratio, and centrifuged at 400× *g* for 20 min. The PBMC fraction was washed twice in saline and resuspended in 1 mL of differentiation medium consisting of alpha-MEM (pH 7.4, Gibco™) supplemented with 10% fetal bovine serum (FBS, Gibco™), 25 ng/mL human M-CSF (Gibco™), 50 ng/mL human RANKL (Thermo Fisher, Waltham, MA, USA), 5 ng/mL human TGF-β1 (Gibco™), and 1 µM dexamethasone (Gibco™, Waltham, MA, USA).

Cell viability and concentration were determined in a Neubauer chamber using Trypan Blue (1:1 dilution). For osteoclast differentiation assays, PBMCs were seeded at a density of 6 × 10^5^ cells per 1.9 cm^2^ in 200 µL of differentiation medium. Cultures were maintained for 15 days, with 50% of the medium replaced twice weekly.

### 4.2. Model Clarification

This model utilizes PBMCs as a source of osteoclast (OC) precursors. Supplementation with M-CSF and RANKL promotes osteoclastogenic differentiation while maintaining a heterogeneous cell population that includes other monocyte-derived lineages, such as macrophages. This cellular heterogeneity more closely reflects the in vivo OC microenvironment, where precursor cells interact with multiple mononuclear populations. Consequently, while our transcriptomic analysis predominantly captures the signature shifts associated with osteoclastogenic differentiation, it also includes contributions from unfused mononuclear and macrophage-like cells. Rather than a limitation, this model provides a broader, more physiologically relevant representation of the cellular interactions that govern osteoclast biology and bone remodeling processes.

### 4.3. Determination of Non-Cytotoxic Concentrations

Non-cytotoxic concentrations of *B. moojeni* venom and its fractions were established based on previously published protocols [8]. Crude venom was pooled from six adult specimens (three males, three females) maintained under controlled conditions at the Center of Excellence for the Discovery of Molecular Targets (CENTD). To obtain high HMM and LMM fractions, the crude venom (10.0 mg/mL) was processed using a 10 kDa molecular mass cut-off membrane. Fraction composition and separation efficiency were validated via mass spectrometry. Viability assays confirmed that concentrations of 5 µg/mL for crude venom and HMM and 1 µg/mL for LMM exhibited no significant cytotoxicity toward OC precursors, consequently, these specific doses were selected for all subsequent osteoclast differentiation experiments [5].

### 4.4. Total RNA Isolation

On day 15 of differentiation, OCs were harvested using a cell scraper and homogenized in 100 µL of TRIzol. Following a 10-min incubation at room temperature, the mixture was centrifuged at 12,000× *g* for 15 min at 4 °C to induce phase separation. The upper aqueous phase, containing the RNA, was transferred to a 1.5 mL tube to which 1 µL of glycogen and 250 µL of isopropanol were added. After gentle inversion and a 10-min incubation, the RNA was precipitated following centrifugation at 12,000× *g* for 30 min at 4 °C. The resulting pellet was washed three times with 1 mL of 90% ethanol, followed by centrifugation at 12,000× *g* for 20 min. Finally, the pellet was air-dried for 5–10 min and resuspended in 100 µL of nuclease-free water. This study employed three independent biological replicates (*n* = 3) per experimental group for subsequent transcriptomic analysis.

### 4.5. RNA Integrity Analysis

RNA integrity was assessed using the Agilent 2100 BioAnalyzer system (Agilent Technologies), which employs microfluidic gel-based electrophoresis and laser-induced fluorescence. The system calculates the RNA Integrity Number (RIN) based on electropherogram profile, focusing heavily on the integrity of the 18S and 28S ribosomal RNA (rRNA) peaks. All samples were strictly required to meet a minimum RIN threshold of 7.0 to ensure high-quality, non-degraded RNA, thereby establishing a rigorous and reliable foundation for the subsequent transcriptomic profiling.

### 4.6. Library Preparation and Sequencing

RNA library construction and high-throughput sequencing were performed by CD Genomics (Shirley, NY, USA). High-quality cDNA libraries were prepared using standard Illumina library preparation protocols for total RNA (mRNA and lncRNA), incorporating universal paired-end adapters. Sequencing was executed on the Illumina platform—generating 150 bp paired-end reads (PE150) to capture both coding (mRNA) and long non-coding (lncRNA) transcripts. Raw sequencing data were securely transferred via encrypted storage to the Butantan Institute, Brazil for subsequent bioinformatic processing using a standardized pipeline for differential gene expression analysis.

### 4.7. Sequencing Quality Control

Raw FASTQ data were evaluated using FastQC (v. 0.11.9). Key metrics included per-base and per-sequence quality scores (Phred scores), GC content balance, and sequence length distribution. The Phred score (Q) was determined using the standard logarithmic scale: Q = −10 X log_10_ (E), where E represents the probability of an incorrect base call. All samples consistently exhibited mean Phred scores greater than 30 (Q > 30, indicating ≥ 99.9% base-calling accuracy), (Table 2) confirming excellent data quality and sequencing depth for downstream differential expression analysis.

### 4.8. Mapping and Read Quantification

Read mapping is a pivotal stage in the RNA-Seq workflow, enabling transcript identification and subsequent assessment of differential expression. In this study, alignment was performed using the STAR aligner (Spliced Transcripts Alignment to a Reference, version 2.7.8a), selected for its high accuracy in detecting splice variants and its efficient use of "soft-clipping" to manage residual adapter sequences and unaligned bases. Reads were mapped to the GRCh38.p13 human reference genome using the corresponding GTF annotation file (release 103) from the Ensembl database to provide the framework for transcript-level annotation. Post-alignment, the resulting BAM (Binary Alignment Map) files were evaluated using MultiQC (version 1.13) to aggregate multiple quality metrics across all samples. Each sample achieved a sequencing depth of at least 10 million mapped reads, satisfying the threshold required for robust downstream expression analysis. Gene-level quantification was subsequently executed using the featureCounts function within the Rsubread Bioconductor package to generate the raw gene expression matrix.

### 4.9. Normalization and Transcriptome Processing

To minimize biological and technical noise, lowly expressed genes were filtered prior to downstream analysis using the proportion-based filtering method implemented in the NOISeq package (v2.40.0). This approach excludes features with negligible expression across conditions, thereby enhancing statistical power and the overall robustness of the dataset. Following filtering, raw counts were normalized using the median-of-ratios method within the DESeq2 package (v1.36.0) to correct for variations in sequencing depth and RNA composition across libraries. Differentially expressed genes (DEGs) were subsequently identified using a generalized linear model framework in DESeq2. Statistical significance was strictly defined using an absolute log_2_ fold change threshold of log_2_ FC ≥ 1 (equivalent to a fold change ≥ 2) and a Benjamini–Hochberg adjusted *p*-value (false discovery rate, FDR) < 0.05.

### 4.10. Functional Enrichment Analysis

Biological processes and molecular pathways were investigated through two complementary strategies: Gene Set Enrichment Analysis (GSEA) and Over-Representation Analysis (ORA). While GSEA evaluates coordinated expression patterns across the entire ranked gene list without requiring arbitrary expression cutoffs, ORA identifies enrichment among statistically significant DEGs.

All ORA evaluations and corresponding graphical pathway representations (Figure 1, Figure 2, Figure 3, Figure 4 and Figure 5) were generated using the PANTHER Classification System (version 19.0). To ensure a comprehensive functional interpretation, pathways were cross-referenced and queried against the KEGG (Kyoto Encyclopedia of Genes and Genomes), Reactome, and WikiPathways databases.

## 5. Conclusions

**Figure 6:**
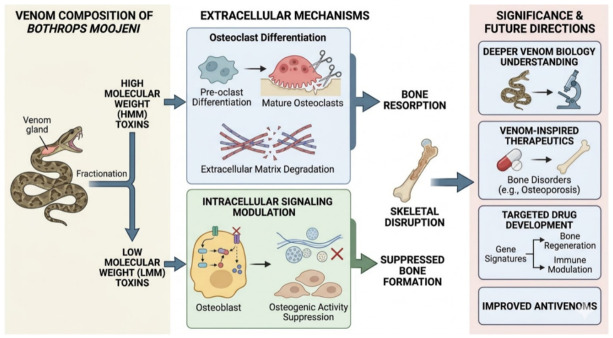
**Differential Effects of *Bothrops moojeni* Venom Fractions on Osteogenic Cells.** This figure illustrates the distinct biological activities of HMM and LMM fractions of *Bothrops moojeni* venom on osteogenic cells. HMM components, including metalloproteinases and serine proteases, promote extracellular matrix degradation, enhance osteoclast differentiation, and alter cytoskeletal structure. In contrast, LMM components, such as phospholipases A_2_ and small peptides, modulate intracellular signaling, suppress osteogenic activity, and regulate gene transcription. The synergistic and complementary actions of these venom fractions underscore their therapeutic potential for modulating bone remodeling and immune function, offering promising avenues for treating bone diseases like osteoporosis and informing venom-inspired drug DEVELOPMENT. Image generated by Gemini, 2026.

This study demonstrates that distinct toxin classes within *Bothrops moojeni* venom are associated with the differential modulation of osteoclastogenic and bone remodeling-related pathways. The HMM fraction was predominantly associated with pathways involved in extracellular matrix remodeling, intracellular trafficking, and inflammatory signaling, whereas the LMM fraction exhibited a more selective profile linked to immune regulation, metabolic homeostasis, and signaling modulation (Figure 6). These findings expand the current understanding of venom-associated molecular responses and provide evidence for the functional compartmentalization of venom components. Importantly, this work represents a comprehensive transcriptional evaluation of fraction-specific venom effects during osteoclastogenic differentiation, offering a valuable framework for future studies exploring venom-derived bioactive molecules with potential applications in bone remodeling, osteoimmune regulation, and related pathological conditions.

## Figures and Tables

**Table 1:** Summary of Fraction-Specific Molecular Effects and Pathway Modulation.

Venom Sample	Key Molecular Effects	Affected Pathways	Unique Features
**Crude Venom (CV)**	Broad activation of neuroimmune, apoptotic, and neurodegenerative pathways Generalized detoxification and cell growth response	Neuroimmune signaling, apoptosis, detoxification, and cell growth	Systemic impact with diverse cellular stress responses.
**High Molecular Mass (HMM)**	Enrichment of cargo receptor activity Modulation of neuroendocrine and protein turnover	Intracellular trafficking, neuroendocrine signaling, and protein turnover	Specific targeting likely driven by unique enzymes and neurotoxins.
**Low Molecular Mass (LMM)**	Selective depletion of homeostatic and reproductive functions Retention of proliferative signaling Modulation of immunomodulatory pathways	Immune regulation, reproductive signaling, and proliferative signaling	Peptide-driven effects, consistent with disintegrins and cysteine-knot peptides.
**Shared Effects (All Groups)**	Consistent suppression of ATP-dependent activities and ligand binding Disruption of basal metabolic and signaling functions	Metabolic pathways Signaling cascades	Indicative of venom-induced cellular stress and metabolic alteration.

**Table 2:** Statistical correlation table.

Phred Score (Q)	Error (E)	Accuracy (1 − Error)
10	1/10 = 10%	90%
20	1/100 = 1%	99%
30	1/1000 = 0.1%	99.9%
40	1/10,000 = 0.01%	99.99%
50	1/1,000,000 = 0.001%	99999%
60	1/10,000,000 = 0.0001%	999999%

## Data Availability

All relevant data are within the manuscript and its Supplementary Materials.

## Supplementary Materials

The following supporting information can be downloaded at: https://www.mdpi.com/article/10.3390/ijms27135943/s1.
