# Supplementary material for: Transcriptomic Dissection of Bothrops moojeni Venom Reveals Fraction-Specific Modulation of Host Cellular Pathways
Source: Int J Mol Sci. 2026 Jul 1;27(13):5943. doi: 10.3390/ijms27135943 (PMC13361672; doi:10.3390/ijms27135943)
Supplement: Supplementary file 1 [file ijms-27-05943-s001.zip › ijms-4248341-supplementary.pdf]

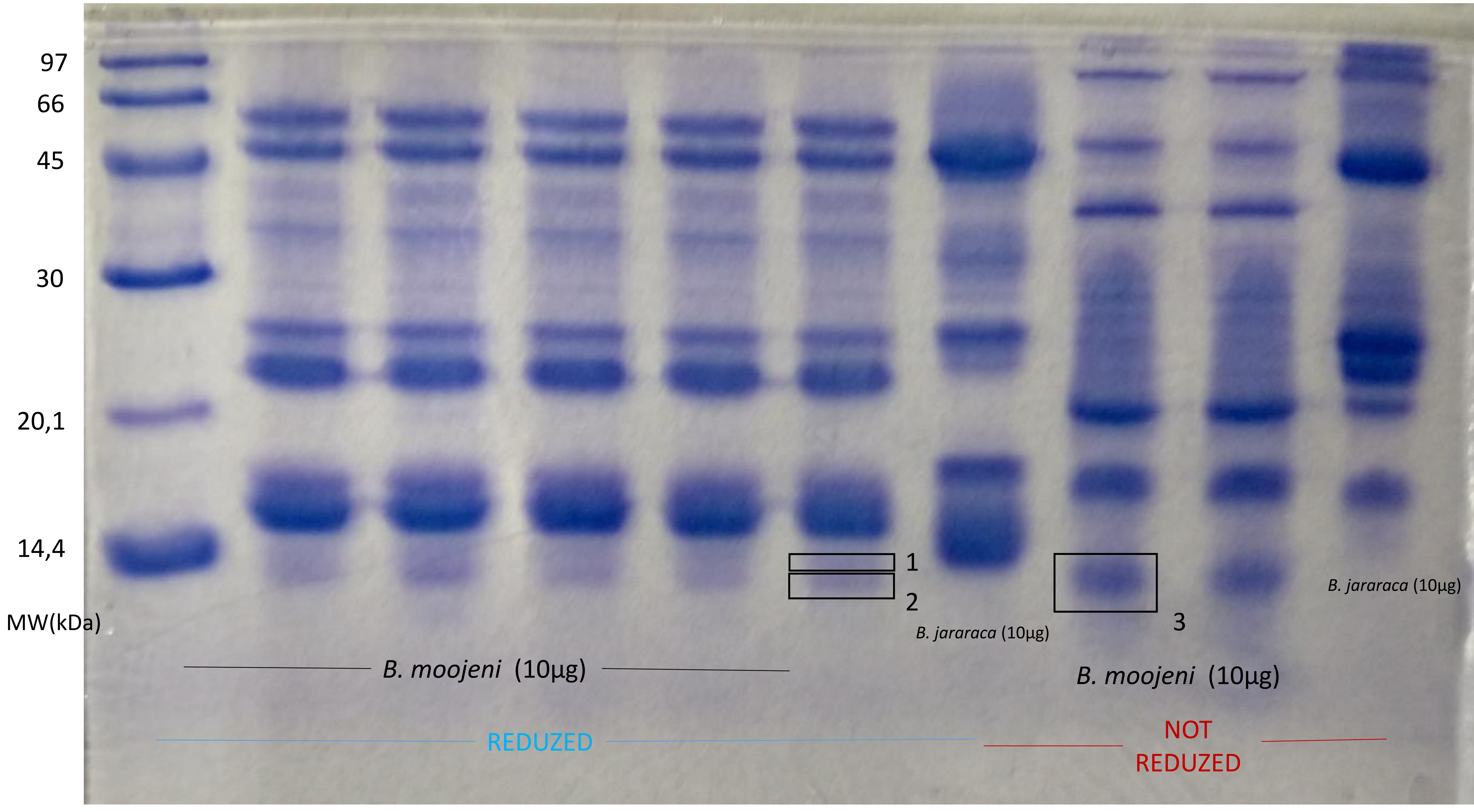
97
66
45
30
20,1
14,4
MW(kDa)
1
2
3
B. jararaca (10µg)
B. jararaca (10µg)
B. moojeni (10µg)
B. moojeni (10µg)
REDUZED
NOT REDUZED

Banda 1	
Coverage	Proteínas identificadas
111.82	Chain A, Crystal Structure Of Bmoompalpha-I, A Non-Hemorrhagic Metalloproteinase Isolated From Bothrops Moojeni Snake Venom
82.58	Zinc metalloproteinase/disintegrin OS=Bothrops asper PE=2 SV=1
82.58	type II metalloproteinase OS=Bothrops asper
82.05	Chain A, High-Resolution Crystal Structure Of The P-I Snake Venom Metalloproteinase Bap1 In Complex With A Peptidomimetic: Insights Into Inhibitor Binding
82.05	Chain A, High-Resolution Crystal Structure Of The P-I Snake Venom Metalloproteinase Bap1 In Complex With A Peptidomimetic: Insights Into Inhibitor Binding
82.05	Chain A, High-Resolution Crystal Structure Of The P-I Snake Venom Metalloproteinase Bap1 In Complex With A Peptidomimetic: Insights Into Inhibitor Binding
82.05	Chain A, Amino Acid Sequence And Crystal Structure Of Bap1, A Metalloproteinase From Bothrops Asper Snake Venom That Exerts Multiple Tissue-Damaging Activities.
82.05	Chain A, High-Resolution Crystal Structure Of The P-I Snake Venom Metalloproteinase Bap1 In Complex With A Peptidomimetic: Insights Into Inhibitor Binding
82.05	type I metalloproteinase OS=Bothrops asper
82.05	Snake venom metalloproteinase BaP1 OS=Bothrops asper PE=1 SV=2
81.51	Snake venom metalloproteinase BpirMP (Fragment) OS=Bothrops pirajai PE=1 SV=1
81.51	metalloprotease BOJUMET I, partial OS=Bothrops jararacussu
[illegible]	[illegible]
81.51	Snake venom metalloproteinase BjussuMP-2 (Fragment) OS=Bothrops jararacussu PE=1 SV=1
69.71	Zinc metalloproteinase/disintegrin OS=Agkistrodon contortrix contortrix PE=1 SV=1
69.71	acostatin beta chain OS=Agkistrodon contortrix contortrix
67.07	metalloprotease BOJUMET III, partial OS=Bothrops jararacussu
67.07	Metalloprotease BOJUMET III (Fragment) OS=Bothrops jararacussu PE=2 SV=1
48.47	BATROXRHAGIN OS=Bothrops atrox
28.52	Jararafibrase-3 (Fragment) OS=Bothrops jararaca PE=1 SV=1
28.52	Ca(2+)-dependent type galactoside-binding lectin {N-terminal} OS=Bothrops jararaca, venom, Peptide Partial, 55 aa
28.52	C-type lectin galatrox (Fragments) OS=Bothrops atrox PE=1 SV=1
28.52	C-type lectin BpLec OS=Bothrops pauloensis PE=1 SV=2
28.52	PAL=lectin OS=Bitis arietans=puff-adders, venom, Peptide, 135 aa
28.52	C-type lectin APL OS=Agkistrodon piscivorus piscivorus PE=1 SV=1
28.52	C-type lectin PAL OS=Bitis arietans PE=1 SV=1
28.52	RecName: Full=C-type lectin APL; Short=CTL
28.52	C-type lectin BjL OS=Bothrops jararaca PE=1 SV=2
28.52	C-type lectin OS=Bothrops insularis
28.52	C-type lectin OS=Agkistrodon piscivorus leucostoma PE=2 SV=1
28.52	C-type lectin BiL OS=Bothrops insularis PE=1 SV=1
28.52	C-type lectin OS=Agkistrodon piscivorus leucostoma
28.52	Uncharacterized protein (Fragment) OS=Anolis carolinensis PE=4 SV=1
26.21	unnamed protein product OS=Agkistrodon piscivorus
25.84	Venom toxin OcyC11 OS=Bactrocera cucurbitae
25.84	Venom toxin OcyC11 OS=Bactrocera cucurbitae

Banda 2	
Coverage	Identificação de proteínas
63.54	Snake venom metalloproteinase BmooMPalpha-I OS=Bothrops moojeni PE=1 SV=2
63.54	Snake venom metalloproteinase leucurolysin-A OS=Bothrops leucurus PE=1 SV=2
41.35	hypothetical protein OS=Anguilla anguilla
41.35	Chain A, Crystal Structure Of Vap2 From Crotalus Atrox Venom (Form 2-1 Crystal)
41.35	Chain B, Crystal Structure Of Vap2 From Crotalus Atrox Venom (Form 2-1 Crystal)
41.35	Chain A, The Three-Dimensional Structure Of Bothropasin, The Main Hemorrhagic Factor From Bothrops Jararaca Venom.
41.35	Chain A, Crystal Structure Of Vap2 From Crotalus Atrox Venom (Form 2-5 Crystal)
41.35	Chain B, Crystal Structure Of Vap2 From Crotalus Atrox Venom (Form 2-2 Crystal)
41.35	Chain B, Crystal Structure Of Vap2 From Crotalus Atrox Venom (Form 2-5 Crystal)
41.35	Chain B, The Three-Dimensional Structure Of Bothropasin, The Main Hemorrhagic Factor From Bothrops Jararaca Venom.
41.35	Chain A, Crystal Structure Of Vap2 From Crotalus Atrox Venom (Form 2-2 Crystal)
41.35	SVMP-CohPH-3 OS=Crotalus oreganus helleri PE=2 SV=1
41.35	SVMP-CohPH-3 OS=Crotalus oreganus helleri
41.35	jararhagin, partial OS=Bothrops jararaca
41.35	Zinc metalloproteinase-disintegrin-like jararhagin (Fragment) OS=Bothrops jararaca PE=1 SV=1
41.35	Zinc metalloproteinase-disintegrin-like VAP2A OS=Crotalus atrox PE=1 SV=1
41.35	vascular apoptosis-inducing protein 2A OS=Crotalus atrox
41.35	RecName: Full=Zinc metalloproteinase-disintegrin-like VMP-III; Short=CvvVMP-III; AltName: Full=Snake venom metalloproteinase; Short=SVMP; Flags: Precursor

41.35	catrocollastatin precursor OS=Crotalus atrox
41.35	Zinc metalloproteinase-disintegrin-like VMP-III OS=Crotalus viridis viridis PE=2 SV=1
41.35	Metalloproteinase (type III) 4a OS=Crotalus horridus
41.35	metalloproteinase VMP-III precursor OS=Crotalus viridis viridis
41.35	Zinc metalloproteinase-disintegrin-like OS=Crotalus durissus durissus PE=2 SV=1
41.35	Zinc metalloproteinase-disintegrin-like VAP2B OS=Crotalus atrox PE=1 SV=1
41.35	metalloproteinase VMP-III precursor OS=Crotalus atrox
41.35	Metalloproteinase (type III) 4b OS=Crotalus horridus
41.35	metalloproteinase P-III OS=Crotalus durissus durissus
41.35	bothropasin precursor OS=Bothrops jararaca
41.35	BATROXRHAGIN OS=Bothrops atrox
41.35	Zinc metalloproteinase-disintegrin-like BITM06A OS=Bothrops insularis PE=2 SV=1
41.35	metalloproteinase precursor OS=Bothrops insularis
41.35	Zinc metalloproteinase-disintegrin-like bothropasin OS=Bothrops jararaca PE=1 SV=2
35.50	Metalloprotease BOJUMET III (Fragment) OS=Bothrops jararacussu PE=2 SV=1
29.72	hypothetical protein OS=Anguilla anguilla
29.72	hypothetical protein OS=Anguilla anguilla
29.72	Protein FAM36A-like protein OS=Crotalus horridus PE=4 SV=1
29.72	protein FAM36A-like protein OS=Crotalus horridus
29.72	protein FAM36A-like protein OS=Crotalus horridus
29.72	protein FAM36A-like protein OS=Crotalus horridus
29.72	hypothetical protein L345_12905, partial OS=Ophiophagus hannah
29.72	Uncharacterized protein (Fragment) OS=Ophiophagus hannah GN=L345_12905 PE=4 SV=1
29.72	uncharacterized protein C18orf25-like OS=Crotalus horridus
29.72	Uncharacterized protein OS=Anolis carolinensis PE=4 SV=2
29.72	FAS-associated factor 1 OS=Crotalus adamanteus
29.36	hypothetical protein OS=Anguilla anguilla
29.36	hypothetical protein OS=Anguilla anguilla
29.36	AP-1 complex subunit mu-2 OS=Ophiophagus hannah GN=AP1M2 PE=4 SV=1
29.36	Uncharacterized protein OS=Anolis carolinensis GN=AP1M1 PE=4 SV=1
29.36	PREDICTED: AP-1 complex subunit mu-2 isoform X3 OS=Python bivittatus
29.36	AP-1 complex subunit mu-2-like protein OS=Crotalus horridus PE=2 SV=1

25.69	PREDICTED: kinesin-like protein KIF24 OS=Python bivittatus
25.69	Serine/threonine-protein phosphatase OS=Anolis carolinensis GN=PPEF1 PE=3 SV=2
25.69	Uncharacterized protein OS=Anolis carolinensis GN=DNM1L PE=3 SV=2
25.69	PREDICTED: dynamin-1-like protein, partial OS=Python bivittatus
25.69	Uncharacterized protein OS=Anolis carolinensis GN=GTF3C2 PE=4 SV=2
25.69	PREDICTED: splicing factor 3B subunit 1 isoform X2 OS=Python bivittatus
25.69	PREDICTED: splicing factor 3B subunit 1 isoform X1 OS=Python bivittatus
25.69	Uncharacterized protein OS=Anolis carolinensis GN=SF3B1 PE=4 SV=2
25.48	hypothetical protein OS=Anguilla anguilla
25.48	toxin 2, partial OS=Bothrops jararaca
25.48	toxin 1, partial OS=Bothrops jararaca
25.48	Toxin 1 (Fragment) OS=Bothrops jararaca GN=Tox1 PE=4 SV=1
25.48	unnamed protein product, partial OS=Agkistrodon piscivorus
25.48	Zinc metalloproteinase-disintegrin bilitoxin-1 OS=Agkistrodon bilineatus PE=1 SV=1
25.48	Protein-lysine 6-oxidase (Fragment) OS=Ophiophagus hannah GN=LOX PE=4 SV=1
25.48	Protein-lysine 6-oxidase, partial OS=Ophiophagus hannah
25.48	Uncharacterized protein OS=Anolis carolinensis GN=LOX PE=4 SV=2
25.48	PREDICTED: protein-lysine 6-oxidase isoform X2 OS=Thamnophis sirtalis
25.48	PREDICTED: protein-lysine 6-oxidase isoform X1 OS=Thamnophis sirtalis
25.48	MP_III3 SVMP (Fragment) OS=Bothrops neuwiedi PE=2 SV=1
25.48	MP_III3 SVMP precursor, partial OS=Bothrops neuwiedi

Banda 3	
Coverage	Proteinas identificadas
119.07	Chain A, Crystal Structure Of Bmoompalpha-I, A Non-Hemorrhagic Metalloproteinase Isolated From Bothrops Moojeni Snake Venom
92.78	BATROXRHAGIN OS=Bothrops atrox
81.15	Snake venom metalloproteinase leucurolysin-A OS=Bothrops leucurus PE=1 SV=2
81.15	RecName: Full=Snake venom metalloproteinase leucurolysin-A; Short=Leuc-A; Short=SVMP
81.15	Chain A, Crystal Structure Of Leucurolysin-a Complexed With An Endogenous Tripeptide (qsw).
80.82	nonhemorrhagic metalloprotease MP-II, partial OS=Bothrops
80.82	metalloprotease BOJUMET I, partial OS=Bothrops jararacussu
80.82	Snake venom metalloproteinase BjussuMP-2 (Fragment) OS=Bothrops jararacussu PE=1 SV=1
79.79	RecName: Full=Snake venom metalloproteinase atroxlysin-1; Short=SVMP; AltName: Full=Atroxlysin-I
79.79	Snake venom metalloproteinase atroxlysin-1 OS=Bothrops atrox PE=1
78.10	Chain A, High-Resolution Crystal Structure Of The P-I Snake Venom Metalloproteinase Bap1 In Complex With A Peptidomimetic: Insights Into Inhibitor Binding
78.10	Chain A, High-Resolution Crystal Structure Of The P-I Snake Venom Metalloproteinase Bap1 In Complex With A Peptidomimetic: Insights Into Inhibitor Binding
78.10	Chain A, High-Resolution Crystal Structure Of The P-I Snake Venom Metalloproteinase Bap1 In Complex With A Peptidomimetic: Insights Into Inhibitor Binding
78.10	Chain A, Amino Acid Sequence And Crystal Structure Of Bap1, A Metalloproteinase From Bothrops Asper Snake Venom That Exerts Multiple Tissue-Damaging Activities.
78.10	Chain A, High-Resolution Crystal Structure Of The P-I Snake Venom Metalloproteinase Bap1 In Complex With A Peptidomimetic: Insights Into Inhibitor Binding
78.10	type I metalloproteinase OS=Bothrops asper
78.10	Snake venom metalloproteinase BaP1 OS=Bothrops asper PE=1 SV=2
72.55	Metalloprotease BOJUMET III (Fragment) OS=Bothrops jararacussu PE=2 SV=1
72.55	metalloprotease BOJUMET III, partial OS=Bothrops jararacussu
63.20	Zinc metalloproteinase/disintegrin VMP-II OS=Crotalus viridis viridis
63.20	metalloproteinase VMP-II precursor OS=Crotalus viridis viridis
58.07	unnamed protein product OS=Sistrurus miliarius
41.25	Ht-e, hemorrhagic toxin e, atrolysin e=metalloproteinase OS=Crotalus atrox, venom, Peptide Partial, 291 aa
41.25	metalloprotease PII OS=Crotalus durissus collilineatus
41.25	metalloproteinase P-II OS=Crotalus durissus durissus
41.25	Zinc metalloproteinase/disintegrin OS=Crotalus atrox PE=1 SV=1
41.25	preprometalloproteinase OS=Crotalus atrox
41.25	Metalloproteinase P-II OS=Crotalus durissus durissus PE=2 SV=1
41.25	Metalloprotease PII OS=Crotalus durissus collilineatus GN=MPII
35.42	metalloproteinase precursor OS=Agkistrodon contortrix
35.42	metalloprotease
35.42	Snake venom metalloproteinase ACLH OS=Agkistrodon contortrix laticinctus PE=1 SV=1
34.35	non-hemorrhagic fibrin(ogen)olytic metalloprotease, partial OS=Bothrops neuwiedi
34.35	Snake venom metalloproteinase neuwiedase (Fragment) OS=Bothrops pauloensis PE=1 SV=1
34.35	RecName: Full=Snake venom metalloproteinase neuwiedase;
33.66	MP_III3 SVMP (Fragment) OS=Bothrops neuwiedi PE=2 SV=1
33.66	MP_III3 SVMP precursor, partial OS=Bothrops neuwiedi
33.28	unnamed protein product OS=Agkistrodon piscivorus
28.96	Metalloproteinase OS=Crotalus molossus molossus PE=2 SV=1
